# SAFETY AND EFFECTS OF HEAT-INACTIVATED LACTOBACILLUS PARACASEI D3.5 (LPD3.5) ON MENTAL WELLBEING IN OLDER ADULTS

**DOI:** 10.1093/geroni/igae098.4069

**Published:** 2024-12-31

**Authors:** Amanda Lloyd, Alina Warren-Walker, Nigel Holt, Doug Lynch, Denis Kay, Kevin Stephens, Shalini Jain, Hariom Yadav

**Affiliations:** Aberystwyth University, Ceredigion, Wales, United Kingdom; Aberystwyth University, Ceredigion, Wales, United Kingdom; Aberystwyth University, Ceredigion, Wales, United Kingdom; Postbiotics Inc, Winston Salem, North Carolina, United States; Neurodyn Life Sciences Inc, Charlottetown, Prince Edward Island, Canada; Agroceutical Products Ltd, Glasbury, England, United Kingdom; University of South Florida, Tampa, Florida, United States; University of South Florida, Tampa, Florida, United States

## Abstract

A healthy gastrointestinal (GI) tract and its microbiome are crucial for maintaining overall health, while microbiome abnormalities can lead to various health issues including brain health and impact quality of life (QOL). These issues are often linked to dysregulation of the gut-brain axis. Although probiotics and prebiotics are common strategies to improve gut and brain health, they have limitations. Postbiotics, offering stable and predictable exposure, present an attractive alternative. In this open-label pilot study, we tested PoZibio®, a postbiotic containing heat-inactivated Lactobacillus paracasei D3.5 (LpD3.5), in 13 healthy older adults (≥50 years) over 30 days to assess its safety, tolerability, and impact on GI function and QOL. Participants consumed four LpD3.5/PoZibio capsules daily and completed the SF-36 and GSRS surveys before and after the study. PoZibio was found to be safe and well-tolerated, with notable improvements in emotional well-being and gastrointestinal symptoms. However, due to the study’s open-label design, small sample size, and short duration, these findings should be interpreted cautiously. Larger, placebo-controlled trials are needed to confirm PoZibio’s potential benefits for the gut-brain axis and overall health.

